# Non-disclosure of developmental neurotoxicity studies obstructs the safety assessment of pesticides in the European Union

**DOI:** 10.1186/s12940-023-00994-9

**Published:** 2023-06-01

**Authors:** Axel Mie, Christina Rudén

**Affiliations:** 1 Department of Environmental Science, Stockholm University, Stockholm, 10691 Sweden; 2 Department of Clinical Science and Education, Karolinska Institutet, Södersjukhuset, Stockholm, 11883 Sweden; 3 Centre for Organic Food and Farming (EPOK), Swedish University of Agricultural Sciences (SLU), Ultuna, Uppsala, Sweden

**Keywords:** Developmental neurotoxicity, Non-disclosure, Pesticides, Plant protection products, Regulatory assessment, Reporting bias

## Abstract

**Background:**

In the European Union (EU), the safety assessment of plant protection products relies to a large extent on toxicity studies commissioned by the companies producing them. By law, all performed studies must be included in the dossier submitted to authorities when applying for approval or renewal of the active substance.

**Methods:**

For one type of toxicity, i.e. developmental neurotoxicity (DNT), we evaluated if studies submitted to the U.S. Environmental Protection Agency (EPA) had also been disclosed to EU authorities.

**Results:**

We identified 35 DNT studies submitted to the U.S. EPA and with the corresponding EU dossiers available. Of these, 9 DNT studies (26%) were not disclosed by the pesticide company to EU authorities. For 7 of these studies, we have identified an actual or potential regulatory impact.

**Conclusions:**

We conclude that (1) non-disclosure of DNT studies to EU authorities, in spite of clear legal requirements, seems to be a recurring phenomenon, (2) the non-disclosure may introduce a bias in the regulatory risk assessment, and (3) without full access to all performed toxicity studies, there can be no reliable safety evaluation of pesticides by EU authorities. We suggest that EU authorities should cross-check their data sets with their counterparts in other jurisdictions. In addition, applications for pesticide approval should be cross-checked against lists of studies performed at test facilities operating under Good Laboratory Practice (GLP), to ensure that all studies have been submitted to authorities. Furthermore, rules should be amended so that future studies should be commissioned by authorities rather than companies. This ensures the authorities’ knowledge of existing studies and prevents the economic interest of the company from influencing the design, performance, reporting and dissemination of studies. The rules or practices should also be revised to ensure that non-disclosure of toxicity studies carries a significant legal risk for pesticide companies.

## Background

We have recently reported that a developmental neurotoxicity (DNT) study indicating adverse effects of glyphosate trimesium was performed in 2001, but was never submitted to regulatory authorities in the EU. Withholding data from regulatory scrutiny can introduce a bias in the risk assessment, and hence hinder authorities to reliably pursue a high level of protection of human health as required by the legislation [1].

In the present work, we follow up on this finding, and systematically evaluate if DNT studies of pesticides that have been submitted to the U.S. EPA have, or have not, been disclosed to EU authorities.

## Introduction

### Pesticides, their use and their safety

In this contribution, the term “pesticides” refers to the active ingredients of plant protection products. Such products are used in agriculture to protect crops from weeds, insect pests, and diseases. As most active substances used for this purpose are designed to be toxic to living organisms, their approval is highly regulated and comprises comprehensive testing for their efficacy, toxicity and ecotoxicity.

### Developmental neurotoxicity (DNT)

Brain development is a complex and delicate process during which cells divide, migrate, mature, specialise, interconnect and are weeded out, to form the organs we use for shaping our lives and taking complex decisions. Chemical-induced disturbances of brain development have been shown to lead to diverse consequences such as decrements in Intelligence Quotient (IQ), attention deficits, or Minamata disease in humans [2]. In addition to individual suffering, such disturbances may also have significant socioeconomic consequences. Conversely, prevention of chemical exposures causing brain developmental effects is associated with substantial gains. For example, the phase-out of lead in fuel, paint and other products caused a decrease in human blood lead levels corresponding to a mean increase of 2.2–4.7 IQ points in birth year cohorts of the late 1990s compared to the late 1970s in the US. This translates to economic benefit for each year’s cohort of 110 to 319 billion US$, through increased productivity [3]. In the EU, the exposure to organophosphate insecticides mainly via the diet has been estimated to cost each birth year’s cohort between €46.8 billion to €194 billion in lost IQ points [4]. This estimate was based on exposures measured almost two decades ago; the use of most organophosphates has since been ended in the EU, and exposures have thus likely decreased substantially.

For some compounds, it has taken decades from the initial evidence of DNT effects in humans until such hazard became widely recognised [5].

### Developmental neurotoxicity testing

Technical guidelines for testing chemicals for developmental neurotoxicity have been developed and refined [6–8]. In these standardized tests, groups of female rats are exposed daily to one of several doses of a test compound, or to a negative control, during pregnancy and lactation. The offspring is evaluated for neuropathological and behavioural alterations. Neuropathology includes qualitative lesions (histopathology) as well as quantitative measurements of the size of different brain layers (morphometry). Behavioural functions evaluated include motor activity and its habituation, auditory startle reflex, learning and memory, as well as the ontogeny of at least two behaviours. Official guidance documents aid in the interpretation of the results [9, 10]. DNT testing may also be performed as add-on to reproductive toxicity testing, although this is rare for pesticides [11].

Tests using this paradigm are sensitive to a number of known human developmental neurotoxicants [12, 13], and represent today the main tool available to evaluate DNT of pesticides for regulatory purposes. Limitations include a high economic cost and a significant number of animals needed per compound studied; the small number of evaluated behavioural functions, meaning that significant effects can remain undetected [14]; and difference in the timing of brain developmental events in relation to birth between humans and rats [15]. In some cases, humans have been shown to be substantially more sensitive to administered doses compared to rodents [16]; in such cases the use of animal data will underestimate the risk for humans. In any case, treatment related effects observed in this test system are used as evidence that a compound can disturb neurodevelopment in humans [10, 12, 13]. Thus, DNT studies are considered to provide reliable and relevant information for the pro-active, pre-market safety evaluation of chemicals [17]. According to an estimate from 2020, approximately 165 chemicals have thus far been tested according to one of the DNT test guidelines [18].

### Principles of EU pesticide regulation

It is an explicit purpose of EU pesticide legislation to “ensure a high level of protection of both human and animal health and the environment”. The risk assessment should be based on the collective evidence, and in general, it aims to establish that the proposed use does not have any harmful effects for human health nor any unacceptable effects on the environment, within the scope of the tested hazards and the foreseen exposures. For certain types of serious effects, legal hazard-based cut-off criteria are in place. If such a criterion is fulfilled, a non-approval is normally triggered, based on the pesticide’s inherent hazardous properties, irrespective of exposure [19]. Aims, principles and details of pesticide safety evaluations differ between jurisdictions and depend on the politically determined level of protection to be achieved. For example, the hazard-based cut-off criteria are unique to the EU.

The overall role of the authorities is to oversee, evaluate and draw the final scientific conclusions based on the data and assessments provided by the pesticide producer. The EU Commission decides on the approval or non-approval based on these conclusions.

Pesticide companies are responsible for providing sufficient documentation. Such documentation consists to a large degree of the results from studies that have been funded by the applicant(s). Some companies run tests at their own testing facilities, but typically, a company commissions the required toxicity studies to an external test laboratory that performs the test, analyses and interprets the data, and writes a study report. As many elements of the safety evaluation of pesticides are in the hand of the companies seeking approval, trust that companies act responsibly is a legally codified characteristic of this process.

### Practices of EU pesticide regulation

The approval of active substances in the EU, and the authorisation of plant protection products containing such active substance in EU member states, are highly formalised and regulated processes. Important elements, with relevance for the present work, include, in chronological order:

- One or more companies apply for EU approval or renewal of the active substance by submitting a “dossier”, i.e. assessments, summary documentation and detailed study reports regarding the safety and efficacy of their substance.
- A Rapporteur Member State (RMS), together with a co-RMS, evaluates the dossier and writes an Assessment Report, which may be updated several times during an evaluation process.
- EFSA organises a consultation where the public, the applicants, the member states, and EFSA itself can comment on the assessment report.
- EFSA organises an expert meeting with their own and member states’ experts.
- EFSA publishes their conclusions of the assessment. At the same time, the peer review report (PRR) is published, which documents the consultation and expert meeting.
- Separate from EFSAs activities, the Risk Assessment Committee (RAC) of the European Chemicals Agency (ECHA) evaluates if hazard classification according to the Regulation on classification, labelling and packaging (CLP) [20] is warranted. This evaluation is based on the Assessment Report.
- The EU Commission passes legislation regarding the (non-)approval or (non-)renewal of the active substance. The EU Commission needs support by a qualified majority of member states for the decision.
- Upon application from a producer, national authorities evaluate if plant production products containing the active substance can be authorised for use in that country, and if any restrictions to its use should apply.

In each of these steps, authorities may draw conclusions and make interpretations that differ from the applicant’s original view.

### All performed studies must be submitted to authorities

In two different circumstances, companies need to inform authorities of toxicity studies they have performed: First, during the approval process of the active substance as part of the dossier, and second, whenever new relevant knowledge is gained, member states must be informed.

Data requirements, i.e. a definition of the information that companies need to include in a dossier for the approval of active substances, have been governed by EU legislation for three decades [21–23]. According to these, there is no general requirement to perform a DNT study. However, it may be required “when indicated by observations in other studies or the mode of action of the test substance” [23].

Nevertheless, there is an unconditional requirement that information about studies conducted must be reported in the dossier. Accordingly, if a DNT study has been performed, it must be included in the dossier even if it is not a part of the standard test requirements. As a minimum, a justification for not including it must be provided. In addition, there are explicit requirements to (1) include any information on potentially harmful effects of the active compounds, and (2) that the included information shall be sufficient to evaluate risks to humans. This would include study evaluations, made by authorities from other jurisdictions, if these indicate harmful effects. Excerpts from the relevant legislation are shown in Table 1.

**Table 1:** Selected data requirements with relevance for existing DNT studies over time for active substance dossiers submitted to EU authorities

Scope	Directive 91/414/EEC Applicable for dossiers submitted before July 2011	Regulation 544/2011 applicable for dossiers submitted between July 2011 and December 2013	Regulation 283/2013 applicable for dossiers submitted from January 2014
Necessary/ sufficient information for evaluating the foreseeable risks to be included in dossier	[The required information shall] include a technical dossier supplying the information necessary for evaluating the foreseeable risks, whether immediate or delayed, which the substance may entail for humans, animals and the environment and containing at least the information and results of the studies referred to below (Annex II paragraph 1.5)	[The information required shall] include a technical dossier supplying the information necessary for evaluating the foreseeable risks, whether immediate or delayed, which the active substance may entail for humans, animals and the environment and containing at least the information and results of the studies referred to below (Annex paragraph 1.1)	The information shall be sufficient to evaluate the foreseeable risks, whether immediate or delayed, which the active substance may entail for humans, including vulnerable groups, animals and the environment and contain at least the information and results of the studies referred to in this Annex. (Annex paragraph 1.1)
All information on potentially harmful effects to be included	There is a need to investigate and report all potentially adverse effects found during routine toxicological investigations (including effects on organs and special systems such as immunotoxicity and neurotoxicity) and to undertake and report such additional studies which may be necessary to investigate the probable mechanism involved, to establish Noaels (no observed adverse effect levels), and to assess the significance of these effects. All available biological data and information which is relevant to the assessment of the toxicological profile of the substance tested, must be reported. (Annex II Part A paragraph 5 point (ii))	There is a need to investigate and report all potentially adverse effects found during routine toxicological investigations (including effects on organs and special systems such as immunotoxicity and neurotoxicity) and to undertake and report such additional studies which may be necessary to investigate the probable mechanism involved, to establish Noaels (no observed adverse effect levels), and to assess the significance of these effects. All available biological data and information which are relevant to the assessment of the toxicological profile of the substance tested, must be reported. (Annex Part A paragraph 5 point (ii))	Any information on potentially harmful effects of the active substance, its metabolites and impurities on human and animal health or on groundwater shall be included. (Annex paragraph 1.2)
Full and unbiased report of all studies conducted to be included	[The information required shall] include a full and unbiased report of the studies conducted as well as full description of them or a justification, which is acceptable to the competent authority where: — particular data and information which would not be necessary owing to the nature of the product or its proposed uses, are not provided, or — it is not scientifically necessary, or technically possible to supply information and data (Annex II paragraph 1.5)	[The information required shall] include a full and unbiased report of the studies conducted as well as full description of them or a justification, which is acceptable to the competent authority where: — particular data and information which would not be necessary owing to the nature of the product or its proposed uses, are not provided, or — it is not scientifically necessary, or technically possible to supply information and data;	The information shall include a full and unbiased report of the studies conducted as well as a full description of them. Such information shall not be required, where one of the following conditions is fulfilled: (a) it is not necessary owing to the nature of the product or its proposed uses, or it is not scientifically necessary; (b) it is technically not possible to supply. In such a case a justification shall be provided. (Annex paragraph 1.5)
Need to perform a DNT study	(DNT not explicitly mentioned)	(DNT not explicitly mentioned)	When indicated by observations in other studies or the mode of action of the test substance, supplementary studies or information may be required to provide information on the postnatal manifestation of effects such as developmental neurotoxicity. (Annex paragraph 5.6.2 last subparagraph)

Furthermore, after market authorisation of plant protection products, there is a requirement to immediately inform the member states where the products are marketed, of any new information that suggests that the approval criteria may no longer be fulfilled [19, 21]. For example, new information on DNT effects that could lead to a classification as Repr. 1B, hence fulfilling the hazard-based cut-off criterion, or to the lowering of reference values, must be communicated without delay. Excerpts from the relevant legislation are shown in Table 2.

**Table 2:** Requirements over time for submitting new data on active substances to national authorities

scope	Directive 91/414/EEC Applicable before June 2011	Regulation 1107/2009 applicable from June 2011
Company’s action if new studies indicate new effects	Member States shall prescribe that the holder of an authorization or those to whom an extension of the field of application has been granted in accordance with Article 9 (1) must immediately notify the competent authority of all new information on the potentially dangerous effects of any plant protection product, or of residues of an active substance on human or animal health or on groundwater, or their potentially dangerous effects on the environment. Member States shall ensure that the parties concerned immediately notify this information to the other Member States and to the Commission, which shall refer the information to the committee referred to in Article 19. (Article 7)	The holder of an authorisation for a plant protection product shall immediately notify the Member States that granted an authorisation of any new information concerning that plant protection product, the active substance, its metabolites, a safener, synergist or co-formulant contained in the plant protection product, which suggests that the plant protection product no longer complies with the criteria set out in Articles 29 and 4 respectively [*i.e. the approval criteria for plant protection products and active substances*]. (Article 56 paragraph 1)

While outside the scope of the present paper, similar rules regarding the obligation to submit all data and to continuously update the database when facing new relevant information, apply in the EU to chemicals other than pesticides [24, 25].

### Scientific and ethical principles

While it is the responsibility of a company to produce data and perform a risk assessment, it is crucial that regulatory authorities have the possibility to make their own evaluation of the available studies. The importance of considering all available data for an assessment is intuitively clear. Withholding data can distort the knowledge base, leading to biased assessments, wrong decisions and in the worst case, insufficient risk management.

The decision to include a study in the evidence base for an assessment of efficacy or risk should never be dependent on the effects reported in that study. A systematic de-selection (and non-disclosure) of studies based on undesired results will cause a bias in the conclusions. This is sometimes referred to as “cherry-picking” and constitutes one form of reporting bias.

The suppression of product safety related results that are unfavourable to the commercial interests of companies, through non-disclosure to regulators or through avoidance of publication, is a strategy that has been observed in several industries. Often, evidence is revealed during litigation processes in the US [26].

Well-documented historical examples of withholding data and knowledge on significant adverse properties include the cases of PFAS [27] and tobacco smoke [28]. An example of withholding unfavourable efficacy data stems from the drug oseltamivir (Tamiflu). Tamiflu was believed to reduce the risk of serious complications from influenza. However, Roche, the maker of the drug, withheld some of their clinical trial data for several years from independent meta-analyses regarding this outcome. Once those data were made available by the manufacturer and included in an updated meta-analysis, the data did no longer support the claim that oseltamivir reduced the risk for serious complications from influenza [29–31].

As described above, studies should be reported to the authorities regardless of their results. This means that studies that do not show any apparent adverse effects, or only seemingly irrelevant findings, should still be disclosed. The reason for this is that data can become meaningful when put into context with additional, or new, knowledge. In this way, data from a study can include pieces of information that, on themselves, are inconclusive, but that can become meaningful when combined with other data.

An example of this, directly relevant to the current paper, is the hazard assessment of the insecticide active substance pymetrozine: the risk assessment committee (RAC) at the European Chemicals Agency (ECHA) concluded based on three different studies including one DNT study: “In summary, RAC notes an array of developmental effects of minor concern […] that considered individually, would probably not trigger classification. However, considering all these effects together, they demonstrate developmental toxicity potential of pymetrozine” [32].

From a different area of science, another example is the elucidation of the molecular structure of DNA, which was enabled by the integration of several types of seemingly irrelevant evidence; an inference that of course changed biology forever [33].

These perspectives support the requirement in the EU pesticide regulation to disclose all data available, including studies that, when considered in isolation, do not indicate any significant or remarkable adverse effects.

Furthermore, we argue that regulatory science should comply with the common principles of research ethics. This includes truthful and transparent reporting of data, and open discussion of results, including the work of others. Hence, to take actions to suppress information with the intention to affect regulatory decisions in a particular direction is not ethically acceptable. It would be against the general rules and ideals of science, violate the trust society puts in scientists employed by laboratories and pesticide producing companies [34]. And, of course, in the case of withheld DNT studies, potentially jeopardize children’s brain development and their chances to reach their full potential.

In consequence, the consideration and disclosure to authorities of all performed pesticide toxicity studies is not only a legal, but also a scientific and ethical obligation of the applicant company.

## Methods

### Selection of DNT studies

We identified guideline DNT studies submitted to the U.S. EPA from four collections: An academic article from 2009 co-authored by U.S. EPA staff [13]; a collection of studies received by the EPA from 2017 [35]; the ToxRef database version 2.0 from 2019 [36]; and a collection of EPA OPP reviews of DNT studies from 2022 [37]. We also included three additional studies received by EPA but identified from other sources [38, 39].

Only full-scale DNT studies were included, testing a pesticide active substance and performed according to any of the test guidelines issued by the EPA (1991, 1998) or OECD (2007) [6–8]. The test guidelines all prescribe prenatal and postnatal exposure and the evaluation of neuropathological and behavioural outcomes in the offspring.

We did therefore not include

- pilot, range-finding or other preliminary studies.
- positive control studies.
- studies of pesticide metabolites.
- studies that focused on a narrow selection of outcomes (e.g., only cholinesterase inhibition).
- extended one-generation reproductive toxicity studies comprising a “Cohort 2” for evaluating DNT, in accordance with OECD TG 443.
- academic studies from the open literature.

From this initial collection, we then excluded.

- studies of compounds without agricultural applications, which are regulated under a different EU framework than active substances used in plant protection products.
- studies of compounds where no company has ever applied for approval under common EU legislation, as identified in the EU Commissions database of active pesticide compounds [40].
- studies of compounds where the summary dossier was not available from the OpenEFSA portal, e.g. because no company ever applied for an ”Annex I Renewal” (AIR) i.e. re-approval of the active substance, or because dossiers had been withdrawn before a decision on the (re-)approval had been taken.

Finally, duplicate or repeat studies were counted as one, i.e. cases where two studies were performed on the same compound by the same laboratory in close temporal proximity.

### Identification of undisclosed DNT studies

For the selected DNT studies, the most recent EU summary dossier for the corresponding compound was accessed. In cases where the DNT study was included in the summary dossier, no further action was taken. In cases where the DNT study was not included in the summary dossier, we investigated if and under what circumstances the DNT study had subsequently been submitted to regulatory agencies, by accessing additional documents, as needed. Records considered for this purpose included:

- assessment reports (ARs) in all available versions.
- addenda to ARs.
- peer review reports (PRR) that document and resolve the points raised during EFSAs consultation as well as minutes from expert meetings.
- EFSA conclusions on the peer review including their appendices containing lists of endpoints (LoEPs).
- ECHA opinions regarding the classification of compounds according to the CLP including associated and supplementary documents.

In addition, for compounds that are ingredients of at least one plant protection product authorised in Sweden at the time the undisclosed DNT study report was issued, we inquired with the Swedish Chemicals Agency if any holder of a registration had informed them of the existence of that study or its results.

### Assessing the regulatory impact of undisclosed studies

In those cases where EU agencies already had requested access to undisclosed DNT studies and fully taken these into account, we classified the regulatory impact of that DNT study as “yes” if at least one of the following decisions was explicitly partly or solely based on the DNT study:

- setting of the toxicological reference values (ADI, ARfD),
- classification according to the CLP,
- decision regarding non-renewal.

Otherwise, the impact was classified as “no”.

In cases where EU agencies have not yet fully evaluated or considered an undisclosed study, we classified the regulatory impact as “potential” if at least one of the following criteria was met:

- the point of departure for any EU toxicological reference value could plausibly be based on the NOAEL or Lowest Observed Adverse Effect Level (LOAEL) for DNT as identified by the test laboratory, by EFSA or U.S. EPA.
- The DNT study could plausibly contribute to a classification as Repr. 2, Repr. 1B, or Repr 1 A according to the CLP, because offspring developmental effects were observed at a dose that did not cause overt maternal toxicity, as identified by U.S. EPA or EU regulatory agencies. This criterion was also considered to be fulfilled if EPA highlighted but ultimately did not rely on an observed effect.

In this context, the term “potential” was chosen to reflect that a specific legal data requirement is triggered, which prescribes that companies must submit “[a]ny information on *potentially* harmful effects of the active substance” (emphasis added. See Table 1). According to our understanding, the threshold for this trigger must be low, so as to put agencies in a position to evaluate any conclusion drawn by the company. The term is thus not meant to pre-empt or suggest any final conclusion regarding a compound’s properties by agencies.

Otherwise, the impact was classified as “less likely”.

If no evaluation by EU authorities or U.S. EPA were available, the regulatory impact was considered to be “unknown”.

### Limitations

Throughout this paper, we identify companies by short names. We thus do not distinguish between e.g. different national branches of the same corporation. Further, we did not investigate any potential co-ownership or other forms of cooperation between companies or test laboratories, unless this was obvious from the name. In cases where the applicant for EU approval or renewal was different from the study sponsor, we were not in a position to investigate if the non-disclosure of a DNT study was due to a failure of the sponsor to inform the applicant, or due to a failure of the applicant to highlight or submit the study to authorities.

The present article reflects our understanding of how the pesticide regulatory system works and should work, from our perspective as scientists. It should not be read as a detailed legal analysis of any company’s action or inaction.

## Results

### Identification of existing DNT studies with EU relevance

**Fig. 1:**
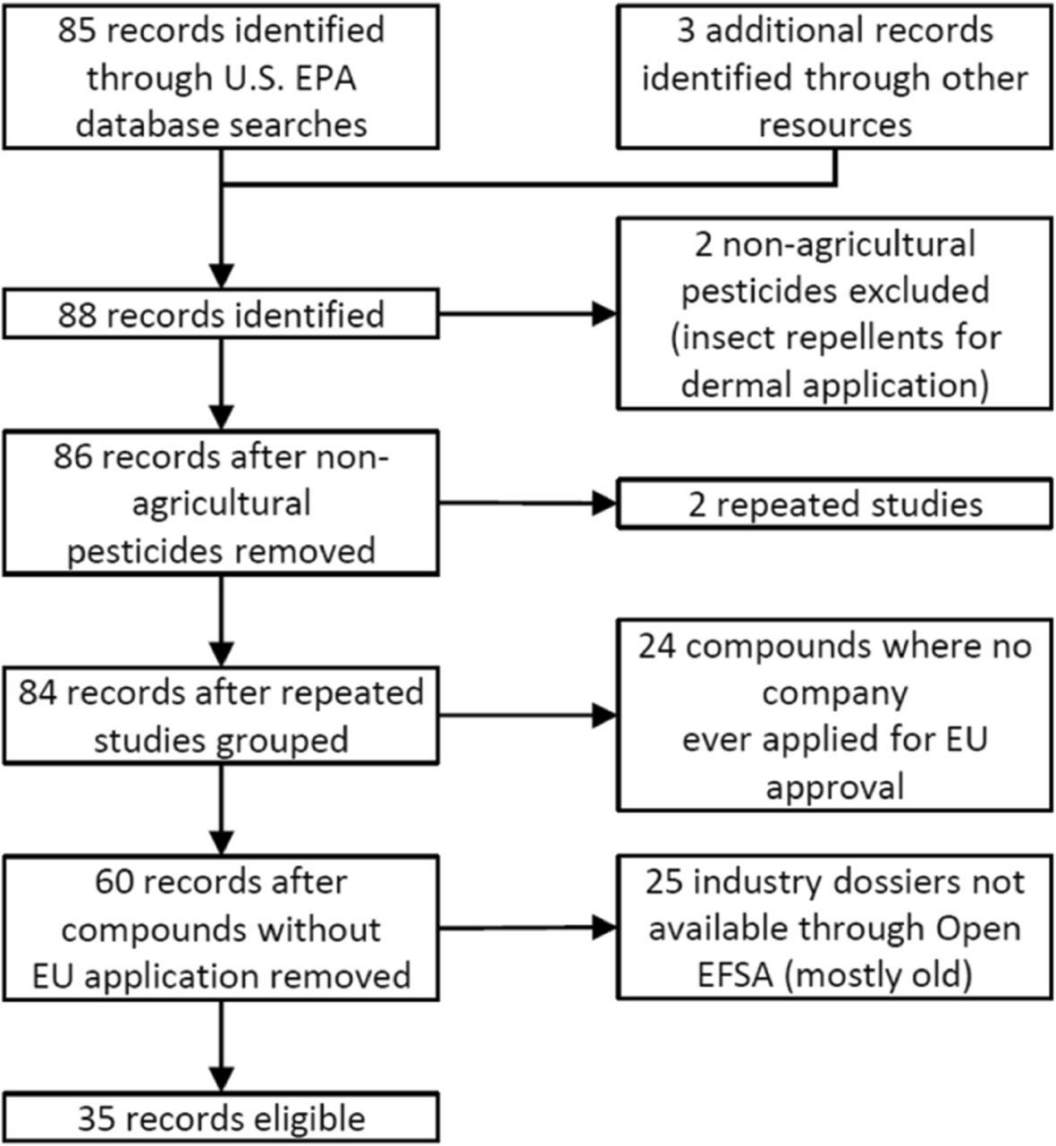
Flow chart of pesticide DNT studies considered for and included in the present work

We have identified 35 DNT studies for pesticide active substances that have been submitted to the U.S. EPA and where summary industry dossiers are available from the OpenEFSA platform in the EU (Fig. 1). These 35 studies form the population included in the present work.

### Identification of undisclosed DNT studies

Of the 35 identified DNT studies, 26 (74%) were included in the most recent corresponding EU summary dossier, while 9 (26%) were not (Table 3). In none of these 9 cases could we find a justification for the non-inclusion. Products containing 4 of the compounds were approved in Sweden at the time the DNT study report was issued. In none of these cases, the sponsor informed the Swedish Chemicals Agency of the DNT study or its results. For the 9 compounds, the results from the DNT study, the EU regulatory history, and the potential for regulatory impact of the DNT study are summarised in Table 4. Findings for each compound are summarised here:

**Table 3:** List of active substances with existing guideline DNT studies included in this work. Dossier submission: year indicated in dossier ”Document A”, or the dossier creation year in IUCLID database, or equivalent, for the most recent EU evaluation available in Open EFSA. In those cases where several applicants have submitted separate dossiers for the same round of evaluation but during different years, those years are all indicated. DNT study report years refer to the original study report, disregarding any amendments

compound name	currently approved (January 2023)	DNT study report year	dossier submission	DNT study included in dossier?
Abamectin	yes	2005/2007	2016	**no**
Acetamiprid	yes	2003	2014	yes
Acibenzolar-S-methyl	yes	2002	2011	yes
Beta-cyfluthrin	no	2003	2014	yes
Boscalid	yes	2001	2016	yes
Buprofezin	yes	≈ 2002	2020	**no**
Chlorpyrifos	no	1998	2015	yes
Chlorpyrifos-methyl	no	2015	2015	yes
Clodinafop-propargyl	yes	2003	2015	yes
Cymoxanil	yes	2001	2019	yes
Deltamethrin	yes	2006	2013/2014	yes
Dimethoate	no	2001	2016	yes
Emamectin benzoate	yes	1993	2022	yes
Ethoprop	no	2004	2016	**no**
Etofenprox	yes	2002	2019	yes
Fenamidone	no	2005	2013	**no**
Fenamiphos	no	2004	2017	**no**
Fluazinam	yes	2005	2016	**no**
Flufenacet	yes	2000	2014	yes
Glyphosate (trimesium)	yes	2001	2021	**no**
Indaziflam	pending	2008	2020	yes
Indoxacarb	no	2006	2015	yes
Isoxaflutole	yes	2000	2013	yes
lambda-Cyhalothrin	yes	2004	2020	yes
Malathion	yes	2002	2019	yes
Mancozeb	no	2008	2015/2018	yes
Mepiquat chloride	yes	2006	2016	yes
Prothioconazole	yes	2004	2015	yes
Pymetrozine	no	2003	2012	**no**
Pyridaben	yes	2007	2020	**no**
Tebuconazole	yes	1998	2017	yes
Thiacloprid	no	2000	2014	yes
Thiram	no	2005	2014	yes
Tri-allate	yes	1998	2019	yes
Ziram	yes	1996	2014	yes

**Table 4:** Summary of undisclosed DNT studies: timeline, study results, and regulatory impact. DER – Data Evaluation Record. PoD – Point of departure. Bw – body weight. f – female, m – male. “---” – we have no access to this information. Laboratories: Bayer: Bayer CropScience LP Toxicology. Stilwell, KS, USA. CTL: Central Toxicology Laboratory, Macclesfield, Cheshire, UK. Huntingdon: Huntingdon Life Sciences, Ltd., Huntingdon, Cambridgeshire, England, UK

	Companies and regulatory timeline					DNT study conclusions							Regulatory impact of DNT study				
							by test laboratory		by agency								
Substance name	Sponsor/ test laboratory	DNT study report	Annex I inclusion, applicants	Applicants Annex I renewal (AIR), dossier submitted	AIR outcome	Dose levels	Off-spring NOAEL^a^	Offspring critical effect^a^	Offspring NOAEL	Offspring critical effect	Mater-nal NOAEL	Source	When/how did EFSA become aware of DNT study?	Most recent PoD (NOAEL) for ADI mg/kg bw/day	Most recent PoD (NOAEL) for ARfD, mg/kg bw/day	Source for PoDs	Regulatory consequences of DNT study
Abamectin	Syngenta/CTL	2005 + 2007	Syngenta 2009	Five companies not incl. Syngenta^b^ 2016	Renewal 2023	0, 0.12, 0.2, 0.4 mg/kg bw/day	---	---	< 0.12	Decreased bw, delayed sexual maturation in f	0.4	EFSA RAR 2020	RMS requested, RAR 2020	0.12 (DNT rat, LOAEL)	0.12 (DNT rat, LOAEL)	EFSA Conclusion 2022	yes
Buprofezin	---/---	approximately 2002	Nihon Nohyaku 2011	Nihon Nohyaku 2020	pending	---	---	---	---	---	---	---	Sept. 2022, by us	0.9 (2-y rat)	50 (developmental rat)	EFSA Conclusion 2010	unknown
Ethoprophos	Bayer/Bayer	2004	Bayer 2007	AMVAC 2016	Non-renewal 2019	0, 3, 30, 180 ppm	3	AChE inhibition	< 3	motor activity PND 17 m	< 3	EFSA final RAR 2018	EFSA found, Peer Review Report 2018	not set - genotox potential	not set - genotox potential	EFSA Conclusion 2018	yes
Fenamidone	Bayer/Bayer	2005	Bayer 2003	Bayer 2013	Non-renewal 2018	0, 60, 250, 1000, 4700 ppm	1000	decreased bw (gain)	1000	decreased bw (gain). *Increased motor activity on PND 13 + 17 in top two dose groups* ^c^	4700	EPA DER	Oct. 2022, by us	not set - genotox potential	not set - genotox potential	EFSA Conclusion 2016	potential: offspring < maternal NOAEL, possible motor activity effects at maternal NOAEL
Fenamiphos	Bayer (?)/ Bayer	2004	Irvita 2007	AMVAC 2016	Non-renewal 2020	0, 2.5, 10, 50 ppm	---	---	10 (2.1 mg/kg bw/day)	RBC ChE inhibition	2.5	EFSA conclusion 2019	public consultation 2018, our comment	0.083 (chronic dog)	0.25 (acute neurotox dog)	EFSA Conclusion 2019	no
Fluazinam	ISK: Ishihara Sangyo Kaisha/ Huntingdon	2005	ISK 2009	Five companies incl. ISK^d^ 2016	pending	0, 2, 10, 50 mg/kg bw/ day	2	decreased bw (gain).	2	decreased bw (gain). Delayed sexual maturation in m. *Decreased non-perfused brain weights m/f in top two dose groups *^c^	50	EPA DER	EFSA or RMS found, included in draft RAR 2021	1 (2-y mouse)	7 (developmental rabbit)	EFSA Conclusion 2008	potential: offspring NOAEL < PoD for ARfD, offspring < maternal NOAEL
Glyphosate (-trimesium)	Syngenta/CTL	2001	Four com-panies incl. Syngenta^e^ 2002	Glyphosate renewal group incl Syngenta and Bayer 2020	pending	0, 10, 25, 100 mg/kg bw/ day	25	decreased survival; bw; memory in high dose m on PND 62	10	decreased PND 14 motor activity in f/m	100	EPA DER, ECHA RAC opinion	March 2022, by us	10 (2-y rat)	150 (developmental rabbit)	RAR 2021	potential: offspring NOAEL < PoD for ARfD
Pymetrozine	Syngenta/CTL	2003	Syngenta (Novartis) 2001	Syngenta 2012	Non-renewal 2018	0, 100, 500, 2500 ppm	100	Mortality, litter size, survival	< 100 (< 8.1 mg/kg bw/day)	brain morphometric changes in f PND 12 and m PND 63 offspring	100	EPA DER	RMS found, approx. 2017	3 (90-d + 1-yr, dog)	10 (developmental rabbit)	EFSA Conclusion 2014	yes
Pyridaben	Nissan Chemical/ Huntingdon	2007	Nissan Chemical 2011	Nissan Chemical 2020	pending	0, 25, 50, 100 ppm	50	decreased bw	25 (= 2.2 mg/kg bw/day)	decreased bw. *Increased PND24 auditory startle pre-pulse inhibition at all dose levels in f/m* ^c^	25	EPA DER	Sept. 2022, by us	1 (2-y rat)	5 (developmental study/maternal, rabbit + rat)	EFSA Conclusion 2010	potential: offspring NOAEL < PoD for ARfD, possible auditory startle effects at maternal NOAEL

^a^This information was extracted from U.S. EPAs data evaluation records.

^b^Industrias Afrasa, Lainco, Probelte, Rotam, Sapec Agro.

^c^ *(italic)* highlighted but not relied on by U.S. EPA.

^d^Finchimica, ADAMA Makhteshim, Cheminova, ISK, Nufarm.

^e^Syngenta, Monsanto, Cheminova, Feinchemie Schwebda

- **Abamectin:** A DNT study was performed in 2005 and repeated in 2007 because the brain morphometry measurements in the original study were deemed unreliable. These studies were not submitted by the sponsor Syngenta to EU agencies before or after the original decision for approval was taken in 2008, or in conjunction with an application for the amendment of conditions of approval in 2013. The renewal dossier was submitted by a group of companies not including Syngenta in 2016; it also lacked the DNT studies, and any reference to their existence. The RMS Austria requested the DNT studies from Syngenta between 2016 and 2018, as described in the draft RAR from 2019. At that time, JMPR and other agencies had already based their chronic reference values on those DNT studies. The approval has been renewed in March 2023.
- The new decreased ADI and ARfD, considered for the renewal decision, are based on effects on delayed sexual development observed in the DNT studies [41]. Certain previous uses, e.g. on apples, may lead to exceedances of the new ARfD and will be limited or no longer authorised.
- In Sweden, the abamectin-containing product Vertimec has been authorised since 2005. The authorisation was withdrawn in April 2023. The holder of the authorisation, Syngenta, has never informed the Swedish Chemicals Agency of the existence of this DNT study, or its results.
- **Buprofezin:** Little is known to us about the DNT study of buprofezin, except that a study report was issued approximately in 2002. The study was not included in the dossier from 2020, and we informed EFSA in September 2022 of its existence. EFSA confirmed to us that the study has subsequently been requested from the applicant company Nihon Nohyaku Co, and that it will be considered in the ongoing re-evaluation of buprofezin. The current approval expires in 2024.
- In Sweden, the authorisation of a product containing buprofezin ended in 2000, i.e., before the DNT study report was issued.
- **Ethoprophos:** A DNT study was sponsored by Bayer and performed in their own laboratory, with a report issued in 2004. According to the U.S. EPA-commissioned evaluation of January 2005, ethoprophos caused behavioural effects at all dose levels tested. Specifically, increased motor activity was observed in male pups in the dosed groups on PND 17, evident as a failure to habituate in some animals; this conclusion had apparently not been drawn in the original study report [42]. We do not know when this evaluation was communicated to Bayer, or when Bayer communicated their summary of this study to EU agencies. Nonetheless, the evaluation of the same study by EU agencies, dated April 2005, did not identify, highlight, discuss or conclude any behavioural effects in offspring at any dose level [43]. The EU approval since 2007 was based on this conclusion [44, 45].
- In 2016, a renewal dossier was submitted by the company AMVAC to EU authorities. Neither the DNT study nor its evaluation by U.S. EPA were included or summarised this dossier. In contrast, the other seven studies of reproductive and developmental toxicity that were submitted and considered ahead of the 2007 EU approval, were all summarised, and their ownership was changed from Bayer to AMVAC.
- In 2017, EFSA identified the lack of the DNT study in the 2016 dossier, and we informed EFSA in September 2017 of the U.S. EPA conclusions from 2005 indicating DNT effects at all dose levels tested [46]. This conclusion was ultimately adopted by EFSA [47] and contributed to the non-renewal decision in 2019 [48].
- EFSA also highlighted that an existing repeat-dose comparative cholinesterase assay (CCA), necessary to conclude on the DNT of ethoprophos, was not available to them, although it apparently indicated a higher sensitivity of the offspring compared to adult animals [47]. Indeed, in 2015, U.S. EPA relied on one acute and one repeat-dose CCA for setting the toxicological reference values [49]; none of these studies were included in the EU dossier from 2016.
- Products containing the substance ethoprophos have never been authorised in Sweden.
- **Fenamidone:** A DNT study was performed and sponsored by Bayer, with a study report issued in 2005. In their evaluation, the U.S. EPA identified no adverse effects in maternal animals. Effects on body weight (gain) in offspring in the highest dose group were identified. An apparent increase of total motor activity at the two highest dose levels in both sexes on PND 13 and 17 was noted, but ultimately not relied upon due to high data variability and lack of statistical significance [37]. Previous two-generation and subchronic neurotoxicity studies in rats indicated effects of fenamidone on brain weight in Sprague Dawley rats. EPA therefore requested a DNT study to be performed in the same rat strain, however Bayer submitted a DNT study in a different rat strain (Wistar). EPA has thus requested parts of the DNT study to be repeated with the originally requested rat strain for the evaluation of effects on brain weight [50]. We were not able to establish if that repeat study has been performed; in any case, it has not been submitted to EU agencies. The DNT study was not included in the dossier from 2013, and was thus not available for the EU evaluation that ultimately resulted in non-renewal for other reasons [51]. We have notified EFSA of the existence of this study in October 2022.
- Products containing the substance fenamidone have never been authorised in Sweden.
- **Fenamiphos:** A DNT study was performed and sponsored by Bayer, with a study report issued in 2004. This report was not included or mentioned in the dossier from 2016; instead, the applicant AMVAC submitted a document requesting to waive any requirement for a DNT study. We highlighted the existence of this study during EFSAs public consultation in 2018. The DNT study was subsequently requested from the applicant, and EFSA concluded an absence of DNT effects in offspring, except for erythrocyte cholinesterase inhibition at the top dose level on PND21. A non-renewal decision was taken in 2020 based on other concerns [52].
- Products containing the substance fenamiphos have never been authorised in Sweden.
- **Fluazinam:** A DNT study was sponsored by Ishihara Sangyo Kaisha Ltd (ISK) and performed by Huntingdon, with a report issued in 2005. The U.S. EPA identified no adverse effects in maternal animals, but developmental effects were identified in the offspring at the top two dose levels, including delayed sexual maturation in males. Another observation highlighted, but not primarily relied on by EPA, was a statistically significant dose-related decrease of mean non-perfused brain weights in adult offspring (PND 66) at the mid and high dose levels in both sexes; means were also decreased at the low dose, but statistically non-significant. Several effects on behavioural functions were also described by EPA. Neither the DNT study nor its evaluation by U.S. EPA were included in the EU dossier from 2016. We informed EFSA of the existence of this study in October 2022; EFSA informed us that the study had already been included in the most recent update of the RAR from 2021 (which is not available to us). The study will thus be taken into account during the ongoing re-evaluation. The current approval expires in 2024.
- In Sweden, several products containing fluazinam are currently authorised, including Shirlan, with ISK being the holder of the authorisation since 2000. No holder of an authorisation has ever informed the Swedish Chemicals Agency of the existence of the DNT study, or its results.
- **Glyphosate (trimesium salt)**: A DNT study was sponsored by Syngenta and performed by the Central Toxicology Laboratory, with a report issued in 2001. U.S. EPA identified effects on offspring motor activity at the top two dose levels, in the absence of maternal toxicity [37]. The study was not included in the dossier for glyphosate in 2021. We have highlighted the existence of this study for EFSA in March 2022, and recently reported details [1]. ECHA has confirmed the adverse effects identified by EPA but states that they could not assess if these effects were caused by the test compound or by impurities [53]. This conclusion was apparently based on a mistaken interpretation of the water content of the test substance as an impurity [54]. RAC also highlighted that this particular glyphosate salt is not sold anymore and had previously been regulated separately from other forms of glyphosate. The observed DNT effects were thus excluded from ECHA’s CLP assessment of developmental toxicity of glyphosate.
- EFSA’s evaluation of this study is not yet public but will be available for the ongoing re-evaluation of glyphosate. The current approval for glyphosate expires in 2023.
- In Sweden, products containing glyphosate-trimesium were authorised until 2007, including Syngenta’s product Avans that was authorised until December 2002. A variety of products containing other forms of glyphosate are currently authorised. No holder of an authorisation has ever informed the Swedish Chemicals Agency of the existence of the DNT study, or its results.
- **Pymetrozine**: A DNT study was sponsored by Syngenta and performed by the Central Toxicology Laboratory, with a report issued in 2003. In 2005, U.S. EPA identified effects on offspring brain morphometry at all dose levels tested. The EPA also identified a dose-dependent increase in pups dying during PND 1–5 at all dose levels compared to control [37]. Neither the DNT study nor its evaluation by U.S. EPA were included in the dossier from 2012; these were thus not available for EFSA’s evaluation of the active substance [55], that was followed by a non-renewal decision in 2018 due to risk for groundwater contamination and adverse effects on endocrine organs across several species [56]. RMS Germany was informed [57] (unclear by whom) of the existence of the DNT study during the subsequent process for classification, and the study contributed to the classification as “Repr. 2” [32].
- In Sweden, the product “Plenum 50 WG” containing pymetrozine was authorised between 2007 and 2019, with Syngenta being the holder of the authorisation until 2018. No holder of an authorisation has ever informed the Swedish Chemicals Agency of the existence of the DNT study, or its results.
- **Pyridaben**: A DNT study was sponsored by Nissan Chemical and performed by Huntingdon, with a study report issued in 2007. The U.S. EPA identified decreased offspring body weight at the two highest dose levels, in presence of similar effects in maternal animals. The EPA also identified, but did ultimately no rely on, increased PND24 auditory startle pre-pulse inhibition at all dose levels tested in both sexes. The DNT study was not included in the EU dossier from 2020. We highlighted the existence of this study for EFSA in September 2022. EFSA responded that RMS will be made aware, so that the study can be included in the ongoing re-evaluation.
- In Sweden, no products containing pyridaben have ever been authorised.

### Assessing the regulatory impact of undisclosed DNT studies

The obligation to submit performed toxicity studies for the EU approval process exists irrespective of any effects observed in those studies. It is nonetheless of interest to understand the actual or potential impact of undisclosed studies on the assessment of the safety of pesticides.

For one compound (abamectin), EFSA has based the ADI and ARfD on the DNT study, which contributes to the restriction of certain previous uses. For four compounds (fluazinam, glyphosate, pymetrozine, pyridaben), the results from the DNT study could potentially affect the ADI and/or the ARfD, because the DNT NOAEL was equal to or lower than the point of departure currently used for deriving these reference values.

For one compound (pymetrozine), the DNT study contributed to a classification as “Repr. 2” according to CLP. For four compounds (abamectin, ethoprophos, fenamidone, fluazinam), offspring DNT effects were observed at dose levels not causing overt maternal toxicity; therefore, these studies could potentially contribute to a classification according to the CLP.

For one compound (ethoprophos), DNT effects contributed to the non-renewal decision [48].

In summary, three undisclosed DNT studies have already had regulatory consequences after they had been requested and evaluated by regulatory agencies (abamectin, ethoprophos, pymetrozine). Four DNT studies have a potential effect on toxicological reference values or hazard classification (fenamidone, fluazinam, glyphosate-trimesium, pyridaben). One DNT study had no regulatory consequences (fenamiphos). For one study (buprofezin), insufficient information was available for assessing a potential regulatory impact.

In six cases, we were able to compare the offspring NOAEL concluded by the test laboratory to the NOAEL concluded by agencies (Table 4). In four of these cases, the NOAEL established by the agency was lower than the NOAEL established by the test laboratory. In one case, the NOAELs were numerically equal, but the agency identified a more serious critical effect to base the NOAEL on. In one case, the NOAELs were equal.

### Sponsors, laboratories, and applicants

Of the nine undisclosed DNT studies, three were sponsored by Bayer and performed in their own laboratory. Three studies were sponsored by Syngenta and performed in their Central Toxicology Laboratory. One study each was sponsored by Nissan Chemicals and Ishihara Sangyo Kaisha (ISK), and these were performed at Huntingdon Life Sciences. For the remaining study, the sponsor and laboratory are unknown to us.

In seven of eight cases where the sponsor was known to us, the sponsor was also among the applicants for initial Annex I inclusion; these dossiers were however not available to us and were not discussed above. All 9 DNT studies discussed above were absent from the corresponding subsequent dossier for Annex I Renewal. Two of these applications each were submitted by Syngenta and Bayer (in one case each as part of a consortium), two by AMVAC, and one application each by Nihon Nohyaku Co., Nissan Chemical, and three different consortia comprising a total of 16 additional companies.

## Discussion

### The problems

We have shown that 26% of the eligible 35 DNT studies have not been disclosed to EU regulatory authorities, in spite of legal obligations. To our knowledge, this is the first attempt to systematically quantify underreporting by pesticide companies for any type of toxicity study.

It should be noted that with our methods, it is not possible to identify DNT studies that have been withheld from both U.S. and EU authorities. Nor to identify DNT studies that have been submitted to the EU but not to U.S. authorities. No attempts were made to include other jurisdictions.

We cannot know the companies’ actual reasons for non-disclosure in the documented cases. We therefore do not know to what extent our results can be generalised to other types of studies.

Hypothetically, if the non-disclosure is driven by an intention to avoid submitting data that would make an approval less likely, then it is conceivable that any study indicating a significant hazard would be at increased risk of non-disclosure. This would apply in particular to studies with endpoints related to the cut off criteria (i.e., carcinogenic, mutagenic, or toxic for reproduction (CMR) classification, including DNT endpoints, and endocrine disruption). There could also be incentives related to the resources spent on testing. Studies that are less resource-demanding are more likely to be repeated, perhaps with slightly differing designs, exploiting the flexibility of test guidelines. This flexibility is originally intended to enable a sensitive test design but can in principle be used for the opposite purpose. Likewise, studies with adverse findings on an endpoint with low statistical power could be at higher risk of being repeated. If more than one study is available for a particular endpoint, then this enables the submission of only a subset of the performed studies while still appearing to fulfill formal data requirements. It is furthermore possible that studies of a type that is not legally required for all active substances, like DNT, are at higher risk of not being submitted; two additional examples are the CCA studies of ethoprophos discussed above.

The problem of non-disclosed data was recognised long ago. Already in 1976, the then-deputy administrator at the U.S. EPA, John Quarles, highlighted in testimony to a subcommittee of the U.S. Congress the possibility that “valid test results indicating dangerous pesticide characteristics may be withheld from EPA” [58]. Nevertheless, neither regulatory practices nor compliance processes in companies are in place that reliably prevent that practice in the EU today.

While we provide a first quantitative estimate for the fraction of undisclosed studies, we are well aware that this estimate is both incomplete and imperfect, and the true magnitude of this problem remains unknown. It appears, however, that non-disclosure is a problem that should be investigated further. And that it is high time to end the possibility to withhold data from regulatory agencies.

### Consequences of undisclosed studies

The non-disclosure of a DNT study indicating risks or hazards directly counteracts the pursuit of a high level of protection of human health, and potentially puts public health at risk. While each safety assessment is based on many studies, the lack of a single study, or even the misleading analysis of a single endpoint in a single study, can affect the overall conclusion (e.g. [59–61]).

The following example illustrates how the non-disclosure prevents authorities from protecting a vulnerable population group: the fungicide fluazinam is among the most widely used pesticides in intensive apple production in South Tyrol in northern Italy [62]. Fluazinam was also among the most frequently detected pesticides in grass samples from children’s playgrounds located in proximity to apple and wine orchards surveyed in this area in 2017 and 2018, apparently in consequence of spray drift, volatilisation, and/or via contaminated dust [63]. In 24 surveyed sites, the compound was detected at seven locations at spring sampling, at twelve locations in summer, and none in autumn and winter. These data suggest a direct (unintended) exposure of residents in an area of intensive use. By not disclosing the DNT study, the company has, since 2005, deprived EU and, in this case, Italian authorities of the possibility to assess if such exposure is safe with respect to resident children’s brain development.

Furthermore, undisclosed information may hamper the development of new test methods. Guideline DNT studies are expensive, require many animals, their scope is limited, and only a minority of pesticides has been tested according to such guidelines. The use of far cheaper in vitro methods has been proposed to complement in vivo testing. Potentially, authorities could fund a DNT evaluation of all approved pesticides using such methods. One important benchmark for the development of such New Approach Methods (NAMs) is their ability to correctly identify known DNT agents, i.e. their sensitivity [64]. Withholding studies that indicate a DNT hazard may therefore negatively affect the possibility to reliably validate future NAM testing paradigms. Similarly, the identification of negative controls for NAMs may be facilitated by the availability of DNT studies not indicating any adverse effects.

A failure to establish regulatory access to undisclosed studies for existing substances may also effectively be a disincentive to develop new substances, because new substances would then be under harder scrutiny.

### The problem will live on

Since March 2021, a new transparency rule introduced into the EU General Food Law requires newly commissioned toxicity studies of pesticides to be notified to EFSA before or at initiation [65]. This rule enables authorities to check if performed studies have been disclosed.

In this way, the practice of non-disclosure of toxicity studies for pesticides is effectively barred in the EU for studies initiated since March 2021, provided that this requirement is strictly enforced. However, the notification requirement lacks a retroactive component. It will not cure any distortions in the evidence base that may already exist, in the form of undisclosed studies initiated before March 2021. A similar situation has previously been identified for underreporting of clinical studies in the pharmaceutical industry [66].

Among the 318 currently approved active substances in the EU (not counting microorganisms, basic and low-risk substances), only 4 have been approved for the first time during the last 5 years [40, 67]. For a renewal of already approved substances, existing studies are generally not repeated. New studies will typically only be performed if new data requirements are introduced (e.g. in connection with expanded evaluation of endocrine disrupting properties), or in case a particular data gap is identified.

That is, for many years to come, the evidence base for pesticide safety evaluation will consist predominantly of toxicity studies performed before 2021. As we have illustrated, this evidence base is incomplete, and its gaps may put public health at risk.

Below, we therefore discuss solutions addressing underreporting of studies initiated before March 2021.

### Solutions

In our opinion, there is no remedy to the current situation short of ensuring that regulatory agencies get access to the full evidence base, including previously undisclosed studies. We propose four concrete actions to achieve this.

### Collaborate across organisations and jurisdictions

A simple initial means to improve the disclosure of studies for EU agencies is to engage in a data exchange with U.S EPA and other relevant authoritative bodies, to identify studies that have been submitted elsewhere but not in the EU, for already approved and pending active substances. However, this would fall short of addressing non-disclosure at the root, because studies that have never been disclosed to any agency cannot be identified in this way.

### Use the GLP system to identify undisclosed studies

Industry-sponsored pesticide toxicity studies are performed under Good Laboratory Practice (GLP). GLP is a set of management principles and tools to ensure the reliability and documentation of such studies [68]. GLP became mandatory for industry-funded regulatory studies in many countries four decades ago, following the discovery of fraud with such testing in the US [69].

The central aim of GLP is to ensure the integrity of individual studies. Other specified aims include [70].

1. To contribute to an evaluation of chemicals “based on safety test data of sufficient quality, rigour and reproducibility”,
2. To avoid duplication of studies,
3. To ensure that non-finalised (terminated) studies still must result in a final report and be archived.

To our understanding, each of these three points indicates that withholding study reports violates the purpose of GLP. In addition, while GLP principles do not suggest a specific mechanism for preventing the non-disclosure of GLP studies to the relevant authorities, we believe that making available all generated results for regulatory purposes is an implicit but self-evident prerequisite for complying with the intentions of the GLP principles: There is little point in inspecting authorities ensuring that laboratories meticulously document each data point in a study, if that study then can be withheld in its entirety from the intended recipient agencies at the discretion of the sponsor.

According to the Aarhus Convention [71], that has been implemented into EU law [72], parties shall ensure that “[p]ublic authorities possess and update environmental information which is relevant to their functions”. To our understanding, this includes an obligation of the signatories to provide regulatory agencies with the necessary tools to ensure that all industry-funded pesticide toxicity studies are disclosed to them, and an obligation of the agencies to use these tools.

We therefore propose to explore the possibility to make use of existing laws and structures around GLP to address underreporting [1]. More specifically, national authorities regularly perform inspections of GLP-accredited test facilities, and one foreseen subject of such inspection are lists of ongoing and finalised studies [73]. Therefore, such lists of studies, possibly covering many years, may already be in the possession of inspecting authorities or could be obtained as part of normal operations. These lists should be cross-checked with lists of studies included in dossiers submitted to EU authorities for pesticide approval. Studies of currently approved pesticides that have been performed but not been submitted should be requested from the sponsor or applicant. This should also include studies that have been performed but terminated before a final study report was issued.

Ultimately, a global effort would be needed in order to identify all performed studies for an individual compound. However, a start with a smaller number of countries or laboratories is meaningful, as this will help to understand the magnitude of the problem of non-disclosure. We suggest that the EU Commission should initiate and coordinate such an exercise in the EU.

### Change the rules so all studies are commissioned by agencies

Notification of company-commissioned studies at initiation, as now required in the EU food law, addresses the issue of non-disclosure for future studies, but not risks for other types of bias. Typically, such studies are performed at the company’s own site or at an external commercial laboratory. In both cases, the test laboratory is well aware of the economic interest of the sponsor, being able to show that the test compound is safe for use. This situation creates a risk for funding bias, i.e. a tendency for design, performance, reporting of results and conclusions of a study to serve the interests of the sponsor, in this case by attenuating any true adverse effects. Examples from DNT testing include the choice of an inappropriate rat strain in the case of fenamidone described above, or misleading reporting of results in the case of chlorpyrifos [59]. Also, in the majority of evaluated cases in the present work, we found that the test laboratories’ conclusions from DNT studies were more favourable to company than the conclusions by authorities.

We therefore strongly recommend that the rules be changed so that toxicity studies must be commissioned by regulatory authorities to commercial test laboratories, with the costs being recovered from the companies applying for approval [1]. This approach may prevent the interest of the sponsor from affecting the outcome of toxicity studies, and the associated challenges to the safety evaluation of pesticides and to public health.

An alternative option is to perform testing at government-run test facilities, while the costs would still be carried by the applicant company. Similar solutions have been proposed for the testing of drug safety and efficacy [74].

### Introduce legal consequences of non-disclosure

In 2012, the drug manufacturer GlaxoSmithKline pleaded guilty to not submitting certain safety data for their diabetes drug Avandia to the U.S. Food and Drug Administration (FDA), and accepted a criminal fine of approximately 243 million US$ for this failure [75]. In contrast, pesticide companies withholding safety data produced before 2021 from EU regulatory agencies do apparently not risk any penalties, besides having to submit their studies in case the non-disclosure is discovered. It is possible that such non-disclosure would be indictable under national law in some EU member states, as foreseen in Article 72 of the relevant EU Regulation [19], but we are not aware of any example.

We suggest that the rules or practices should be revised so that non-disclosure of toxicity studies carries a legal risk for pesticide companies.

A staff report to a subcommittee of the U.S. Congress recommended already in 1976 that “[t]he problem of the falsification and withholding of safety data is one that is best addressed (A) through continual and systematic monitoring of the pesticide companies and the laboratories, and (B) swift and effective sanctions for violations, including criminal prosecution for intentional violations in this area” [76].

## Conclusions

In this contribution, we show that 26% of 35 eligible DNT studies for pesticides were not disclosed to EU regulatory authorities, in spite of clear legal obligations. To our knowledge, this is the first attempt to systematically quantify companies’ underreporting to regulatory agencies for any type of toxicity study. It is not known to what extent this result can be generalized, but apparently non-disclosure is a problem that is not rare.

It is the responsibility of the pesticide industry to ensure the safety of their products, and to submit all performed studies to EU regulatory authorities. Non-disclosure of DNT studies can introduce a bias into the assessment. There can be no reliable safety evaluation of pesticides by EU authorities without full access to all performed toxicity studies. This can lead to situations where there are pesticides on the market that should not be there.

As a first step, we propose that EU agencies cross check studies submitted to them with studies submitted to the U.S EPA and other relevant bodies. In addition, the authorities inspecting GLP-accredited test laboratories should obtain lists of ongoing and finalised studies to be compared to the contents of regulatory dossiers. Thereby non-disclosed data can be identified.

We furthermore recommend that toxicity studies must be commissioned by regulatory authorities to commercial or public test laboratories, while the costs would still be carried by the applicant company in line with the polluter pays principle. This would also prevent biases related to the design, performance, reporting of results and conclusions in industry-funded toxicity studies.

The rules or practices should also be revised so that non-disclosure of toxicity studies carries a significant legal risk for pesticide companies.

Bayer and Syngenta expressed to the EU commission in 2016 with respect to the further development of the EU pesticide regulatory framework that ”[b]oth of our companies operate with the highest standards of stewardship, safety and proper practice and will continue to do so” [77]. Based on our present work, we conclude that it is essential to supplement trust in such assurances with additional, enforceable, mechanisms to make companies comply with legally binding data requirements, in order to ensure the objectivity of the regulatory system and to safeguard human health.

## Data Availability

The dataset supporting the conclusions of this article is available in Tables 3 + 4. EU legislative documents were accessed from https://eur-lex.europa.eu/. Dossiers, peer review reports (PRR) and assessment reports (AR) including addenda were accessed from the Open EFSA portal at https://open.efsa.europa.eu/ or from EFSAs public consultations site at https://www.efsa.europa.eu/en/calls/consultations.
